# Molecular Interactions between Two LMP2A PY Motifs of EBV and WW Domains of E3 Ubiquitin Ligase AIP4

**DOI:** 10.3390/life11050379

**Published:** 2021-04-22

**Authors:** Min-Duk Seo, Seung-Hyeon Seok, Ji-Hun Kim, Ji Woong Choi, Sung Jean Park, Bong-Jin Lee

**Affiliations:** 1 Research Institute of Pharmaceutical Science and Technology (RIPST), College of Pharmacy, Ajou University, Suwon, Gyeonggi 16499, Korea; mdseo@ajou.ac.kr; 2 Department of Molecular Science and Technology, Ajou University, Suwon, Gyeonggi 16499, Korea; 3 College of Pharmacy and Interdisciplinary Graduate Program in Advanced Convergence Technology & Science, Jeju National University, Jeju, Jeju-do 63243, Korea; sseok@jejunu.ac.kr; 4 College of Pharmacy, Chungbuk National University, Cheongju, Chungbuk 28160, Korea; nmrjhkim@chungbuk.ac.kr; 5 Gachon Institute of Pharmaceutical Sciences, College of Pharmacy, Gachon University, Yeonsu-gu, Incheon 21936, Korea; pharmchoi@gachon.ac.kr; 6 The Research Institute of Pharmaceutical Sciences, College of Pharmacy, Seoul National University, Gwanak-gu, Seoul 08826, Korea

**Keywords:** LMP2A, EBV, NMR, AIP4, E3 ubiquitin ligase

## Abstract

Interactions involving Epstein–Barr virus (EBV) LMP2A and Nedd4 family E3 ubiquitin–protein ligases promote the ubiquitination of LMP2A-associated proteins, which results in the perturbation of normal B-cell signaling. Here, we solved the solution structure of the WW2 domain of hAIP4 and investigated the binding mode involving the N-terminal domain of LMP2A and the WW2 domain. The WW2 domain presented a conserved WW domain scaffold with a three-stranded anti-parallel β-sheet and bound two PY motifs via different binding mechanisms. Our NMR titration and ITC data demonstrated that the PY motifs of LMP2A can recognize and interact weakly with the XP groove of the WW2 domain (residues located around the third β-strand), and then residues between two PY motifs optimize the binding by interacting with the loop 1 region of the WW2 domain. In particular, the residue Val15 in the hairpin loop region between β1 and β2 of the WW2 domain exhibited unique changes depending on the terminal residues of the PY motif. This result suggested that the hairpin loop is responsible for additional interactions outside the XP groove, and this hypothesis was confirmed in a deuterium exchange experiment. These weak but wide interactions can stabilize the complex formed between the PY and WW domains.

## 1. Introduction

Epstein–Barr virus (EBV) is present in all populations, infecting > 95% of humans, and EBV infection persists asymptomatically during the life of the host [1]. EBV is known to cause several human tumors, including Burkitt lymphoma, Hodgkin’s disease, and nasopharyngeal carcinoma (NPC) [2,3,4]. EBV-associated gastric cancer can also be caused by EBV infection [5]. EBV might contribute to the development of breast cancer [6], although this is not yet clear. After infection, EBV maintains latency in most cells, and EBV latency is regulated by latent membrane protein (LMP) 2A, which mimics the B-cell receptor (BCR) and perturbs BCR signaling [7,8,9,10,11,12]. The cytoplasmic N-terminal domain of Latent Membrane Protein 2A (LMP2A NTD) can bind to the protein tyrosine kinases Lyn and Syk, which are important signaling molecules involved in the cascade of normal BCR signaling [13,14,15]. This mimicking results in negative regulation of B-cell signaling, which may enable EBV to escape host immunity [11,16,17]. In addition, it has been revealed that LMP2A modulates STAT and NF-kB transcription factor pathways [18,19,20,21] or other cellular signaling proteins [22,23,24], which suggests that the role of LMP2A in infected cells is more complex than previously thought. In addition, LMP2A expressed in T-cells regulates T-cell receptor (TCR) signaling in a very similar fashion to that of LMP2A in B-cells [25]. 

Atrophin 1 Interacting Protein 4 (AIP4) (also named Itch) is a HECT-type E3 ubiquitin–protein ligase and is a member of the neural precursor cell-expressed developmentally downregulated gene 4 (Nedd4) family [26]. Various proteins, including Nedd4, Smurf1, WWP2, AIP4/Itch, and Rsp5p are members of the Nedd4 family. Nedd4 family proteins modulate various cellular processes, such as regulation of transmembrane protein stability, membrane protein signaling, and virus budding from infected cells [27,28]. The domain structure of Nedd4 family members comprises an amino terminal C2 domain, three or four WW domains, and a carboxyl-terminal HECT domain [27,29].

WW domains are small protein modules (typically 35 to 40 amino acids in length) that contain two conserved Trp residues that play crucial roles in the domain structure and function [30,31,32]. WW domains form a three-stranded antiparallel β-sheet and are linked to various catalytic domains such as HECT E3 ubiquitin–protein ligase domains (in Nedd4 family proteins), rotomerase/peptidyl prolyisomerase domains (Pin1), neuronal protein FE65 [33], kidney and brain expressed protein (KIBRA or WWC1) [34], and Rho GTPase-activating protein domains [27]. In addition to these catalytic domains, the WW domain is a major protein–protein interaction module that is widely found in all biological systems. WW domains have been classified into four groups based on their binding to ligands, and the WW domains of the Nedd4 family are included in group 1, which recognizes Pro-Pro-X-Tyr (PPXY or PY motifs; X being any amino acid) [35]. Interactions involving WW and PY motifs have been implicated in several human cellular disorders [36,37,38]. For example, the binding of Nedd4 WW domains with PY motifs of the epithelial sodium channel (ENaC) allows ubiquitination of the channel by the ubiquitin ligase HECT domain, leading to channel endocytosis and degradation. This process is at least partially impaired in Liddle syndrome, causing increased cell surface retention of channels [39,40,41,42].

LMP2A PY motifs selectively bind to the WW domains of ubiquitin–protein (E3) ligases. LMP2A recruits Nedd4-like ubiquitin protein ligases through the PY motifs, leading to the ubiquitination of LMP2A and several LMP2A-associated B-cell tyrosine kinases, such as Lyn PTK. Ubiquitination of these proteins perturbs normal B-cell signaling by rapid degradation of LMP2A-associated proteins [43]. Thus, LMP2A serves as a molecular scaffold to recruit both B-cell tyrosine kinases and ubiquitin protein ligases, disturbing the essential roles of the B-cell receptor [44,45,46]. In addition, it was found in a mouse model of lymphoma that the p29kip1 tumor suppressor is degraded by enhanced MYC expression promoted by LMP2a [47].

We previously reported that residues between two PY motifs of LMP2A may be involved in binding to WW domains directly and/or indirectly [48]. In addition, the solution structure of the WW3 domain of Itch E3 ligase (AIP4) in complex with the PY peptide derived from the N-terminal PY motif of LMP2A was reported [49]. Here, we present the solution structure of the WW2 domain of hAIP4 and the binding mechanisms involving the WW2 domain and two PY motifs.

## 2. Materials and Methods

### 2.1. Sample Preparation

Four constructs from the LMP2A NTD, which contain PY motifs, were prepared. Detailed information regarding the four constructs is as follows, and the names of the constructs are designated arbitrarily. The PY peptide: PPPPY; the linker peptide (from E_61_ to L_96_): the region between the two PY motifs excluding both PY motifs; N-PY (from E_54_ to L_96_): containing the first PY motif (N-terminal PY motif: P_56_P_57_P_58_P_59_Y_60_) and the linker region; C-PY (from E_61_ to P_103_), containing the second PY motif (C-terminal PY motif: P_97_P_98_P_99_P_100_Y_101_) and the linker region. The constructs were prepared using the pTWIN1 vector with N-terminal Ssp DnaB intein fusion, and cloning, expression, and purification were performed as described previously [50]. The gene (114 bp) encoding the WW2 domain of human AIP4 was purchased from GenScript Corporation (Piscataway, NJ, USA). The synthesized DNA was cloned into the pTWIN1 vector and purified using the same procedures [50]. The sequence of WW2 contained the N-terminal GRA tag (Gly_1_, Arg_2_, and Ala_3_) derived from the pTWIN1 vector and an additional glycine residue (Gly5) inserted during cloning. The chemically synthesized PY peptide was purchased from ANYGEN (Kwang-ju, Korea). 

### 2.2. Isothermal Titration Calorimetry

Isothermal titration calorimetry (ITC) was carried out on a VP-ITC instrument (MicroCal, Northampton, MA, USA) at 300 K. Solutions of 30 µM WW2 in the cell were titrated with three kinds of LMP2A constructs (N-PY, C-PY, and linker peptide). The concentrations of the solutions in the injection syringe were 600 µM (N-PY, C-PY) and 470 µM (linker peptide). The solutions for the cell and syringe were thoroughly degassed by stirring under vacuum. In total, 28 injections were performed, and each injection volume was 10 µL (first injection volume was 5 µL), and the spacing between injections was 300 s.

### 2.3. NMR Experiments and WW2 Structure Calculation

All NMR spectra were recorded at 303 K on a Bruker AVANCE 600 spectrometer equipped with a cryoprobe (Bruker, Billerica, MA, USA). Backbone assignments were performed with HNCA, HNCACB, and HNCO and confirmed with HNCACO. Side-chain resonances were assigned with HCCH-TOCSY, HBHACONH, 3D ^15^N-TOCSY-HSQC, and CCCONH-TOCSY. Slowly exchanging amide proton and ring proton resonances were assigned by dissolving the protein in D_2_O and acquiring 2D-NOESY spectra. Chemical shifts were referenced externally to the DSS. The overall secondary structure was predicted from the CSI [51] and NOE patterns. The distance restraints for the structure calculation were collected from 3D ^15^N-NOESY-HSQC and ^13^C-NOESY-HSQC by manual and automatic assignments for which CYANA 2.0 was used [52]. Dihedral angle restraints were calculated from chemical shifts using TALOS [53]. Only “Good” predictions in the result by TALOS were used. The “Good” match means that there is no outlier among best 25 database matches. The structures were initially generated with CYANA 2.0, and then refined through standard annealing and torsion angled dynamics using the program CNS 1.1 [54]. The program MOLMOL [55] and Pymol [56] were used to visualize the results of the 20 energy-minimized conformers. The quality of the final structure was analyzed using PROCHECK-NMR [57].

### 2.4. NMR Titrations of WW2 with Various LMP2A Constructs

The conventional 2D-[^1^H,^15^N]HSQC spectra of the [U-^15^N] WW2 domain titrated with various unlabeled LMP2A constructs were obtained at 303 K on a Bruker DRX 500 spectrometer. The WW2 domain and added constructs were dissolved in 50 mM sodium phosphate (pH 6.0), 100 mM sodium chloride, and 7% D_2_O. Unlabeled LMP2A constructs (N-PY, C-PY, linker peptide, and PY peptide) were added to the 50 µM [U-^15^N] WW2 domain at various molar ratios. The spectra were processed and analyzed using NMRPipe/NMRDraw [58] and analyzed using th NMRview program [59]. The chemical shifts of the WW2–LMP2A complexes were compared with the chemical shifts of the free protein in solution. The average chemical shift differences, Δδave for the WW2 backbone amide (^1^H and ^15^N) upon binding the LMP2A constructs were calculated using Δδ_ave_ = [(Δδ^2^_HN_ + Δδ^2^_N_/25)/2]^1/2^ where Δδ_HN_ and Δδ_N_ are the amide proton and nitrogen chemical shift differences, respectively [60]. 

The dissociation constant (k_d_) for the WW2–PY peptide complex was determined from Δδ_ave_ by an iterative nonlinear least squares analysis using the program DataFit (Oakdale Engineering, Oakdale, PA, USA). Titration data were analyzed assuming that the observed chemical shift perturbation is a weighted average between the two extreme values corresponding to the free (Δδ = 0) and the bound state (Δδ = Δδ_max_) such that

(1) $$\Delta \delta /\Delta \delta \max=\frac{\left(A_{0}+B_{0}+Kd\right)-\sqrt{{\left(A_{0}+B_{0}+Kd\right)}^{2}-4A_{0}B_{0}}}{2}$$

where A_0_ and B_0_ are the total molar concentrations of the WW2 and the PY peptides, respectively. 

The ^1^H-^15^N heteronuclear NOE experiment was performed under the same conditions. A total of 1024 data points and 128 increments were measured in the direct and indirect dimensions. The amide–proton exchange (H/D exchange) experiment was started immediately after the addition of D_2_O to a sample of ^15^N-labeled proteins (apo WW2, WW2/N-PY, and WW2/C-PY complexes) lyophilized from the buffer. ^1^H-^15^N HSQC spectra of the proteins dissolved in D_2_O were obtained at 288 K on a Bruker DRX 500 spectrometer.

### 2.5. Protein–Peptide Docking

To obtain the modeled structure of the WW2 domain–PY motif complex, the docking process was performed with the solution structure of the AIP4 WW2 domain (PDB ID: 2kyk) that was determined in this study, and the structure of the peptide (EEPPPPYED) that was extracted from the complex structure involving Itch WW3 domain and PY-peptide (PDB ID: 2JOC). The GRAMM-X program was used in this process, following the authors’ guidelines [61].

## 3. Results

### 3.1. WW2 Domain Binding Properties of Two LMP2A PY Motifs

**Figure 1:**
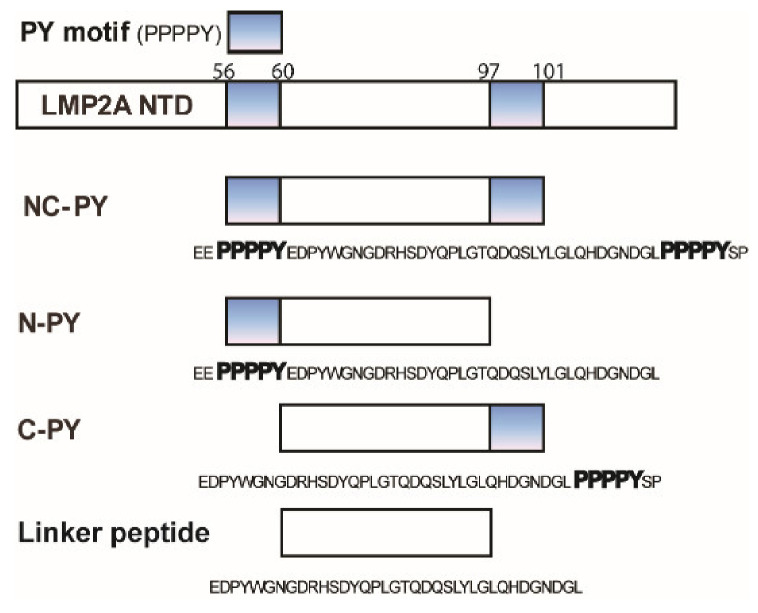
Schematic representation of LMP2A constructs used in this study. The numbers represent the residue numbers of the protein. For N-PY, C-PY, and linker peptide, the exact amino acids sequences are represented.

To identify whether each of the two PY motifs has different binding specificity for WW domains, NMR titration experiments and ITC studies were conducted. In particular, the experiments focused on the importance of expanded regions of the PY motif in binding. We employed the AIP4 WW2 domain and several peptides derived from the N-terminal domain of LMP2A for these interaction studies. The peptides used in this study are presented in Figure 1.

**Figure 2:**
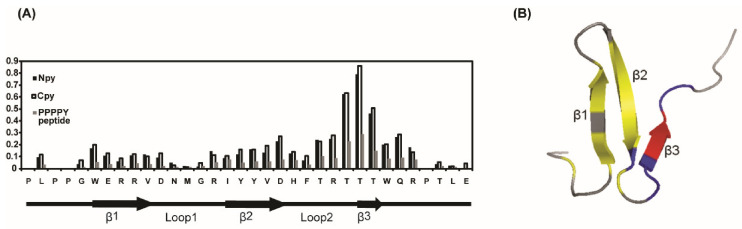
Spectral perturbation of the WW2 domain upon PY motifs binding. (A) Average chemical shift differences (Δδ_ave_) for the WW2 domain backbone amide (^1^H and ^15^N) upon binding to LMP2A constructs (WW2:LMP2A constructs = 1:4). CSP values were calculated according to the formula Δδ_ave_ = [(Δδ^2^_HN_ + Δδ^2^_N_/25)/2]^1/2^. (B) Mapping of the perturbed residues by the LMP2A constructs (N-PY and C-PY) binding. Strong chemical shift changes (Δδ_ave_ > 0.5 ppm) are indicated by red color, and medium (0.2 < Δδ_ave_ < 0.5) and weak (0.1 < Δδ_ave_ < 0.2) chemical shift changes are colored blue and yellow, respectively.

The chemical shift perturbation (CSP) of the WW2 domain following the addition of the unlabeled LMP2A peptides was monitored by recording a series of ^1^H-^15^N HSQC spectra (Supplementary Materials Figures S1–S3). The average chemical shift differences (Δδave) for the WW2 backbone amide upon binding to LMP2A constructs are plotted in Figure 2. As shown in Figure 2, most resonances of the ^1^H-^15^N HSQC spectra of the WW2 domain were perturbed upon binding with the LMP2A peptides, and the changed patterns were similar to those constructs (PY peptide, N-PY, and C-PY, Figures S1–S3). This result indicated that the binding core is conserved in all the complexes between WW2 domain and LMP2A peptides.

**Figure 3:**
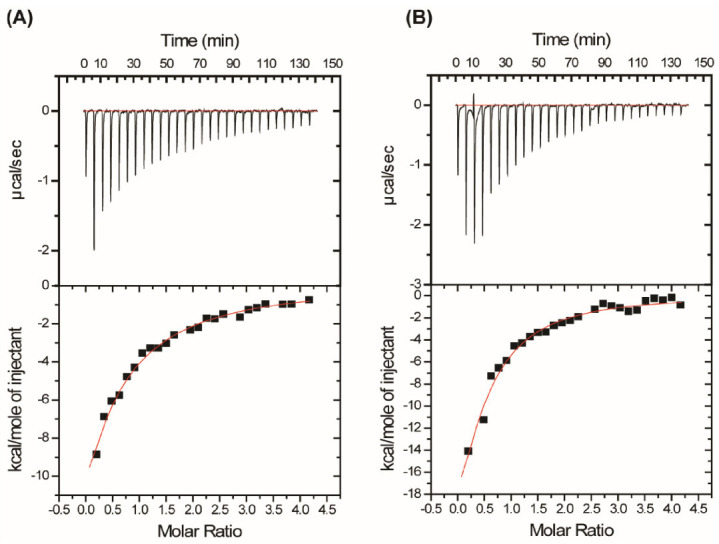
Isothermal titration calorimetry of WW2 domain binding to LMP2A constructs. An amount of 30 µM WW2 (in 50 mM sodium phosphate (pH 6.0), 100 mM NaCl, 1 mM EDTA) was titrated with 600 µM N-PY (**A**), and 600 µM C-PY (**B**), respectively. The best-fitting curve was obtained at the 1:1 binding model.

In the cases of N-PY and C-PY peptides, the three consecutive threonine residues in the third β-strand of the WW2 domain (Thr_30_, Thr_31_, and Thr_32_) were highly affected at relatively low molar ratios, and the changes were almost saturated at a molar ratio of 1:4. In addition, the residues around the third β-strand (Thr_28_, Arg_29_, Trp_33_, Gln_34_, and Arg_35_) experienced large perturbations in their chemical shifts. Some residues in the first and second β-strands were also perturbed. The peaks in the ^1^H-^15^N HSQC spectra of the WW2 domain are continuously shifted during the course of the titrations for all LMP2A peptides. This indicates that exchange is rapid (picoseconds to nanoseconds) in the chemical shift time scale. To determine the binding affinity of the WW2 domain for the two LMP2A peptides (N-PY and C-PY), ITC experiments were performed. Figure 3 shows the titration isotherm of WW2 by N-PY and C-PY, and these curves were fitted using a single binding site model. The dissociation constants (K_d_) for the interaction between WW2 domain and N-PY, WW2 domain and C-PY were 56.23 µM ± 11.6 and 22.61 µM ± 4.8, respectively. The affinity of the WW2 domain for C-PY was approximately two times higher than that for N-PY. The NMR K_d_ values were similar to that of ITC results; the calculated K_d_ values for N-PY and C-PY were 41.19 µM ± 1.91 and 15.30 µM ± 6.34, respectively.

Basically, the synthesized 5-mer PY peptide (PPPPY) exhibited low affinity for the WW2 domain because the CSP values affected by the PY peptide were much smaller than those affected by the other peptides flanking the linker region at the same molar ratio. Most of the WW2 domain resonances did not show significant changes due to PY peptide binding, even at a molar ratio 1:32 (WW2:PY peptide). However, the residues around the third β-strand (Tyr_22_, Tyr_23_, Val_24_, Asp_25_, His_26_, Thr_28_, Arg_29_, Thr_30_, Thr_31_, Thr_32_, Trp_33_, Gln_34_, and Arg_35_) showed meaningful spectral changes compared to other residues (Figure 2A). This result indicated that the region around the third β-strand of WW2 is the major binding site for the PY motif. The binding constant of the PY peptide (PPPPY) was determined from the chemical shift changes obtained from the NMR titration experiment. The Kd value of the PY peptide was 389 µM, which was higher than that of N-PY or C-PY. 

However, the addition of the linker peptide (the linking region between two PY motifs) did not perturb any resonances in the NMR spectrum of the WW2 domain (data not shown), and the titration of WW2 domain by the linker peptide did not induce any detectable heat change in the ITC experiment neither. For further analysis, we also added an excess of the linker peptide (800 µM) into the fully saturated WW2–PY peptide complex (50 µM:800 µM) and measured the ^1^H-^15^N HSQC spectrum. As a result, there were no additional peak changes upon addition of the linker peptide to the complex (data not shown), which indicated that the bonded N- or C-ends of the PY motif are required for accurate binding.

These results revealed not only the major binding site of the WW2 domain, but also the importance of the expanded region of the PY motif, since PY motifs with linker region exhibited a significant increase in binding affinity. Although the residues between the two PY motifs affected WW2 domain binding, it seems that this region could not bind to the WW2 domain by itself.

### 3.2. Solution Structure of the WW2 Domain

**Figure 4:**
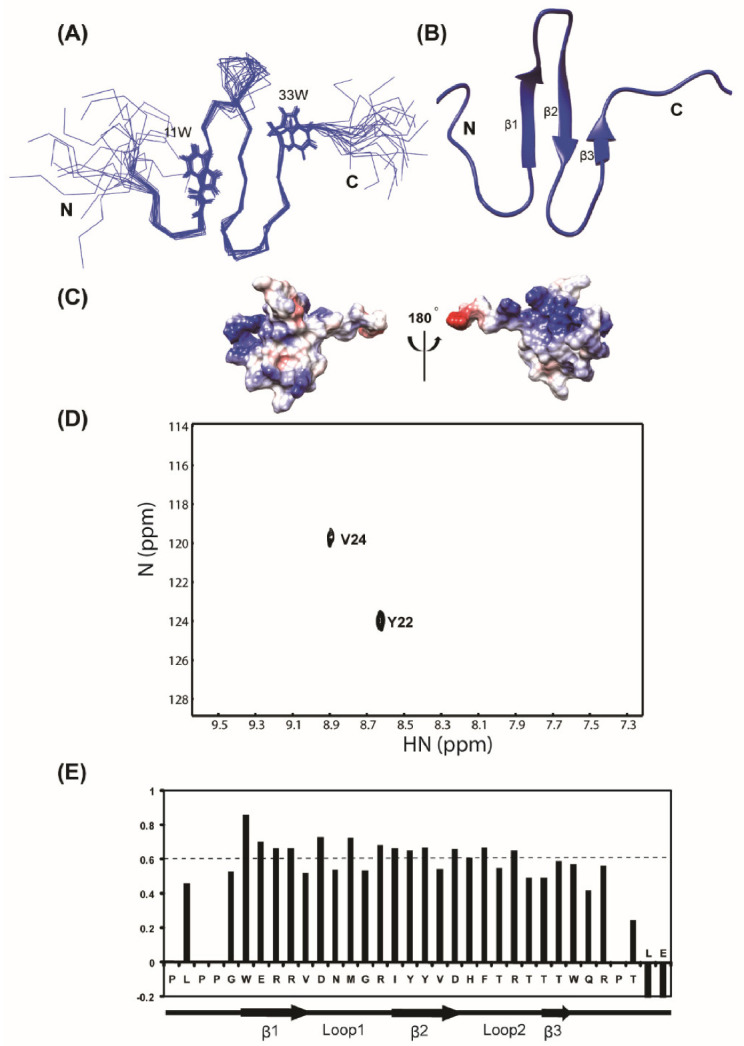
NMR solution structure of the hAIP4 WW2 domain. (**A**) The 20 conformers with the lowest energy are shown after superposition of backbone atoms of residue 7–34 (PDB ID: 2kyk). (**B**) Ribbon drawing of the representative conformer of WW2. (**C**) Surface distribution of charged residues. Positive-charged residues are blue, and negative-charged residues are red. (**D**) ^1^H-^15^N HSQC spectra of the ^15^N-labeled WW2 domain in 100% D_2_O. The spectra were obtained 15 min after the samples were dissolved in D_2_O. The total measurement time for each spectrum was 30 min. (**E**) Backbone flexibility of the WW2 domain. ^1^H-^15^N-Heteronuclear NOE values were plotted as a function of residue number.

The backbone amide (^1^H and ^15^N) resonances of the hAIP4 WW2 domain were completely assigned, except for the four prolines. All carbon resonances (C_α_, C_β_, and CO) were also assigned. H_α_ and H_β_ resonances in the protein were completely assigned, except for one isolated proline, N-terminal methionine, and glycine. In addition, the assignments of the side-chain C and H resonances were almost complete. Based on the NOE data, structural calculations of the WW2 domain were performed. The 20 final structures were well converged with a root-mean-square deviation of 0.39 Å for backbone atoms and 0.99 Å for all heavy atoms of the ordered regions (PDB ID:2kyk). Statistics for the calculated structures are listed in Table 1. As expected, the structure of the hAIP4 WW2 domain is similar to the known WW domain structures, which form a three-stranded anti-parallel β-sheet. The β-strands correspond to residues 11–15 (β1), 21–25 (β2), and 31–32 (β3). The two conserved tryptophan residues are located in the starting position of β1 (Trp_11_) and the position followed by β3 (Trp_33_) (Figure 4A). Unlike other structures of homologous WW domains, the third β-strand (β3) seems to be flexible because the result of the deuterium exchange experiment showed no hydrogen bonding between β2 and β3 (Figure 4D). The heteronuclear NOE experiment also supported that the strand β3 is not well ordered; the NOE values of Thr_31_ and Thr_32_ were below 0.6, while other β-stranded regions presented ≥0.7 NOE values (Figure 4).

### 3.3. Modeled Structure of WW2 Domain–PY Motif Complex

To analyze complex formation, we attempted to obtain intermolecular NOEs between WW2 domains and N-PY or C-PY. However, the long, non-structured tail of the PY motif showed many overlaps of signals, which made NOE analysis very difficult. As an alternative method, we modeled the complex structure of the WW2 domain and PY peptide based on the WW3 domain–PY peptide (EEPPPPYED) complex structure previously published [49]. 

**Figure 5:**
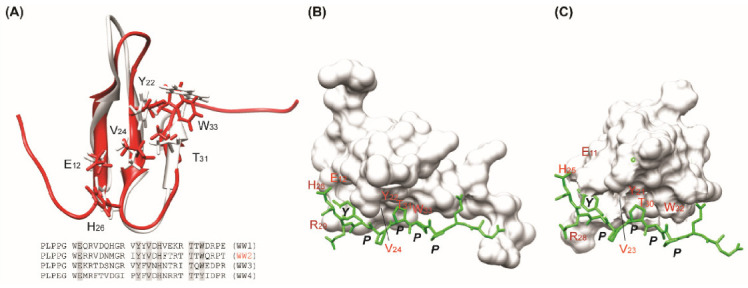
Structural comparison between WW2 and WW3 domains. (**A**) WW2 domain (red) is superimposed on WW3 domain (gray). The six residues mainly required for the PY motif binding are depicted. Four AIP4 WW domains show high sequence homology between the domains. At the bottom of Figure 5A, the conserved residues located in the PY motif binding interface are colored gray. (**B**) The modeled complex structure of WW2–PY peptide (-EEPPPPYED-). The six residues compared in (**A**) are depicted on the structure. The modeling was conducted using the program Gramm-X [61]. (**C**) The reference complex structure of WW3–PY peptide [PDB ID: 2JO9, ref. [49]].

The AIP4 WW2 domain has high structural similarity with the AIP4 WW3 domain as well as high sequence homology. As shown in Figure 5, the overall topology shares similarity except for the N- and C-termini and the residues involved in PY motif interaction are well conserved. For example, the important residues in the WW3 domain (Glu_11_, Tyr_21_, Val_23_, His_25_, Thr_30_, and Trp_32_) that closely interact with the PY motif are well conserved and match those of the WW2 domain (Glu_12_, Tyr_22_, Val_24_, His_26_, Thr_31_, and Trp_33_). In addition, the side-chain orientations of each residue are almost similar. This result implies that the WW2–PY peptide complex is similar to the WW3–PY peptide complex. Therefore, the modeled structure of the WW2 domain–PY peptide could provide reasonable insights into the interactions involved. 

Figure 5B illustrated the modeled complex structure of the WW2–PY peptide. The overall ternary structure of WW2–PY peptide is almost identical to that of WW3–PY peptide (Figure 5); Pro4 of the PY peptide mainly contacts Trp_33_ and Pro_5_ contacts Tyr_22_ and Thr_31_ as in the WW3–PY complex. Tyr_7_ of the PY peptide is also located between His_26_ and Arg_29_. This model structure is consistent with chemical shift changes. Based on this model, the binding mechanism of the short PY peptide may be elucidated to be similar between the WW2 and WW3 domains.

### 3.4. Differences Between Two Tailed PY Motifs Upon Binding

**Figure 6:**
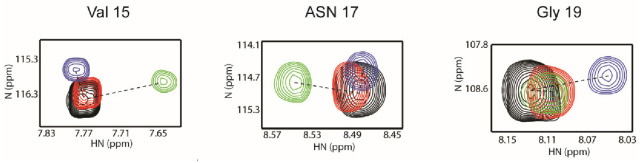
Spectral changes to Val_15_, Asn_17_, and Gly_19_ resonances upon binding PY peptide, N-PY, and C-PY. The superimposed ^1^H-^15^N HSQC spectra of the WW2 domain in the apo-form (black), the PY peptide bound form (red), the N-PY bound form (green), and the C-PY bound form (blue). ^1^H-^15^N HSQC spectra of WW2 were obtained in the presence of 4 molar equivalents of N-PY and C-PY, respectively. The resonances of WW2–PY peptide were obtained at the molar ratio 1:32 (WW2:PY peptide).

It is known that the WW2 domain binds to both PY motifs with similar affinity [44], which is largely consistent with our results. However, the NMR titration data provided details of the binding mode between WW2 domain and two PY motifs. As described above, the overall chemical shift changes in the WW2 domain by N-PY and C-PY peptides appear similar in distance and direction. It is notable, however, that resonances from residues in loop 1 (Val_15_, Asn_17_, and Gly_19_) exhibited somewhat different changes. The cross-peak of Asn_17_ moved only in the N-PY titration, whereas the cross-peak of Gly_19_ highly moved only in the C-PY titration (Figure 6). In particular, the changes in the directions of Val_15_ were quite different in both titrations. The directions of the peak movement of Val_15_ in the N-PY and C-PY titrations were nearly perpendicular (Figure 6). It is well known that chemical shift changes are highly sensitive to the surrounding chemical environment. Thus, the different patterns of chemical shift changes involving residues in loop 1 reflected that this region experienced different surroundings upon binding. Although the CSP values of these residues were smaller than those of other residues in the XP groove, loop 1 seems to be responsible for distinguishing between the two PY motifs. 

**Figure 7:**
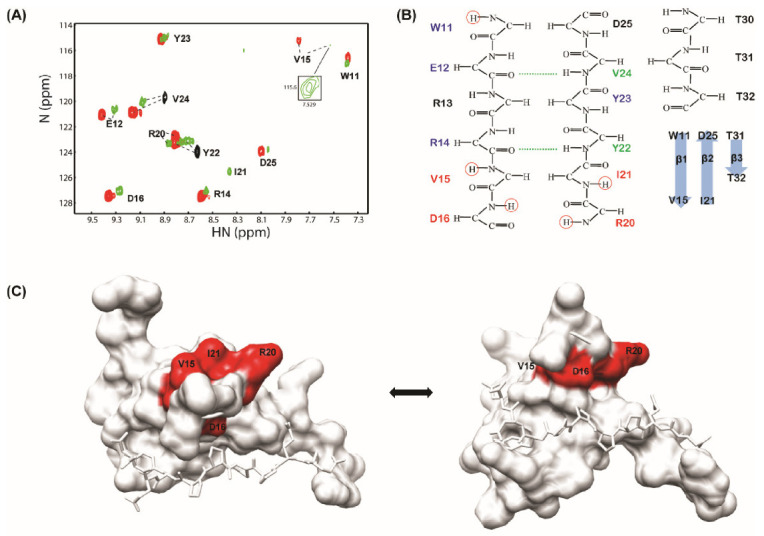
Amide proton exchange (H/D exchange) experiments involving the WW2/N-PY and WW2/C-PY complexes. (**A**) Superimposition of ^1^H-^15^N HSQC spectra of the uncomplexed WW2 domain (black), the bound form of N-PY (green), and the bound form of C-PY (red) in 100% D_2_O at 288K. The molar ratio of the complexes was 1:4 (WW2:ligand). (**B**) Hydrogen bonding network of the WW2 domain. Main-chain hydrogen bonds of the apo WW2 domain are depicted as dashed lines. Four residues showing unexpected protection are colored red. (**C**) Four residues, Val_15_, Asp_16_, Ile_21_, and Arg_20_ are depicted on the WW2–PY peptide complex (red).

This result was also supported by a deuterium exchange experiment. In the free form of the WW2 domain, only Tyr_22_ and Val_24_ were highly protected by deuterium exchange, which indicated that the hydrogen bond network between the β-strands of the WW2 domain was not easily detected in the apo-state. However, the N-PY/WW2 and C-PY/WW2 complexes showed that the amide hydrogens of Trp_11_, Glu_12_, Arg_14_, Val_15_, Asp_16_, Arg_20_, Ile_21_, Tyr_22_, Tyr _23_, Val_24_, and Asp_25_ were protected from solvent exchange upon complexation (Figure 7). This suggests that, in the presence of the N-PY or C-PY peptides, major protection was shown in the β1 and β2 stands of the WW2 domain consisting of the binding interface of the PY motif. Interestingly, the end residues of β1 and β2 stands, Val_15_ and Ile_21_ whose amide hydrogens are not involved in the hydrogen bond network between strands and exposed to the surface of the backside of the PY motif binding interface, were protected in the complexes (the slow exchange of Ile_21_ was only detected in the WW2/N-PY complex). In addition, residues in hairpin loop 1, Asp_16_ and Arg_20_ exhibited slow exchange with deuterium (Figure 7). Thus, this protection pattern may also indicate that an additional interaction occurs between the terminal residues of the PY motif and the loop 1 region.

## 4. Discussion

Many studies regarding interactions between WW domains and PY motifs have been conducted, but most structural studies have focused on the binding of short PY motifs with WW domains [49,62,63,64,65]. The current study may expand our understanding of the binding mode of long-tailed PY peptides and emphasize the effect of the expanded region outside the PY motif upon binding.

### 4.1. Importance of Linker Region Outside of PY Motif

Based on the modeled complex, the core binding interface of the PY motif in the WW2–PY peptide complex showed topological similarity to that of the WW3–PY peptide complex. Compared to the WW3–PY complex, the binding interface of the WW2 domain, namely the XP groove, has a similar sequence composition and provides a similar open space for binding to the PY motif. However, even though the complex structures are similar, the K_d_ values of WW2/N-PY, C-PY, or PY and WW3/PY peptides showed significant differences (the current result and [49]). The tailed PY motif (N-PY and C-PY) in our study showed higher affinity for the WW2 domain than the short PY peptide. Interestingly, the linker peptide between PY motifs did not reveal any evidence of binding by itself (refer to the Results section), which suggested that the PY motifs and the linker peptide should be covalently bonded for optimal binding.

Considering that the major interaction surface of the WW domains (i.e., the XP groove) are well conserved, these differences imply that there is another determinant for regulating binding except for the XP groove. In other words, this may suggest that the area outside the core XP groove may play an important role in binding to the peptide containing the PY motif and may determine specificity for the PY motif. This hypothesis could be supported by our result of deuterium exchange experiments and the difference in chemical shift changes in loop 1. Besides the residues involving the XP groove, residues Val_15_, Glu_16_, Ile_20_, and Arg_21_ of the loop 1 region were additionally protected upon complexation. The chemical shift changes in Val_15_, Asn_17_, and Gly_19_ indicated that loop 1 experienced different chemical surroundings depending on the species of PY peptides involved. A recent study also showed that the phosphorylation of residues outside of the PY motif affected the binding affinity of Nedd4 WW domains by up to two-fold [66].

There are several reports that the WW domain additionally binds to residues outside of the PY motif. The complex structure of the Smad7 PY motif-containing peptide and Smurf2 WW3 domains revealed that six residues flanking the C-terminus of the PY motif bind to the first and second β-strands and the first loop, and some mutations involving these residues reduced the binding affinity for the WW3 domain [62]. This kind of interaction was also shown in the solution structure of the Nedd4 WW3 domain complexed with the Comm PY motif; the flanking residues to the N- and C-termini of the PY motif interacted with the WW3 domain. These contacts are important for affinity and specificity in binding [67]. These results emphasize the importance of loop1 (or β1/β2 loop) in binding with the PY motif, as the residues in the β1/β2 loop are less conserved and are likely to be responsible for interactions with the residues expanded from the C-terminus of the PY motif, which may explain the differences in specificity of Smurf1 and 2 [62]. The interactions between loop 1 and PY peptides have also been observed in other cases, such as the FBP11 WW1–PPLP ligand complex [68] and Nedd4 WW3 domain–Comm PY motif [67]. In addition, loop 1 of the Pin WW domain is intrinsically flexible, and Arg_17_ in loop 1 is responsible for ligand recognition [63,69].

Taken together, it is proposed that PY motifs of LMP2A can recognize and weakly interact with residues spatially located around the third β-strand (XP groove) of WW domains, and then, the linker region optimizes the binding by contacting other residues around loop 1 of WW domains. These weak but wide-scale interactions can stabilize the complex formed between PY motifs and WW domains.

### 4.2. Models of AIP4 and LMP2A Interaction

**Figure 8:**
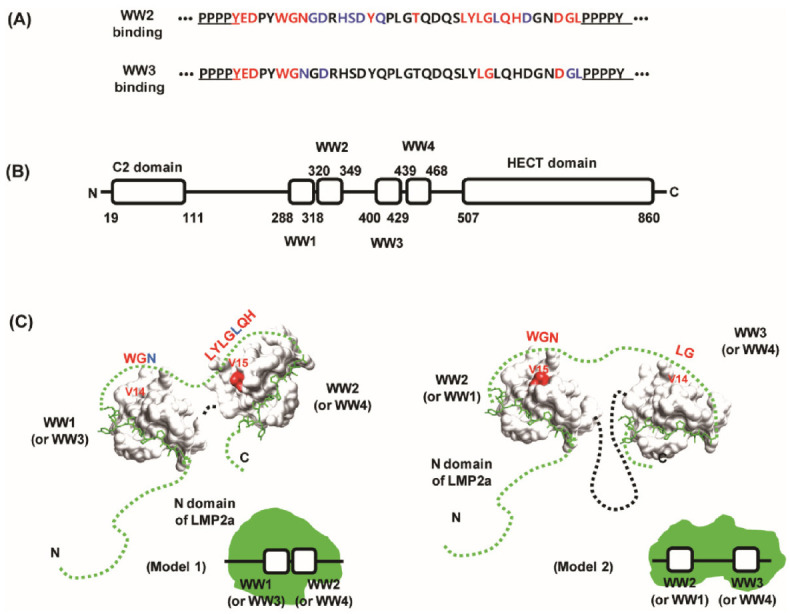
Models of interaction modes involving AIP4 and LMP2A. (**A**) Effects of WW domain binding on the ^1^H-^15^N HSQC spectrum of LMP2A NTD [48]. Upon the binding of WW domains, the resonances from the red colored residues completely disappeared in the two-dimensional ^1^H-^15^N HSQC spectra of the LMP2A NTD, and those of the blue colored residues broadened and/or shifted. (**B**) Domain composition of human AIP4. Residue numbers are depicted on the schematic drawing. (**C**) Two models of interactions. Model 1 represents the consecutive WW domains binding LMP2A NTD. Model 2 represents an alternative way. The potential interaction residues in the linker region are depicted in red color (disappeared in (A)) or with blue color (shifted in (A)). The residue Val in loop 1 of the WW2 or WW3 domain is depicted in the figure.

Previously, we showed that the linker region between two PY motifs of LMP2A NTD was highly affected by WW domain binding (Figure 8A) [48]; upon WW2 domain binding, the peaks of six residues (Tyr_60_, Glu_61_, Asp_62_, Trp_65_, Gly_66_, and Asn_67_) that follow the first PY motif (N-terminal PY motif: P_56_P_57_P_58_P_59_Y_60_) and three residues (Asp_94_, Gly_95_, Leu_96_) that precede the second PY motif (C-terminal PY motif: P_97_P_98_P_99_P_100_Y_101_) disappeared. In addition, the peaks corresponding to Tyr_74_, Thr_79_, Leu_84_, Tyr_85_, Leu_86_, Gly_87_, Gln_89_, and His_90_ also disappeared, indicating that these residues are involved in WW2 domain binding. In the case of WW3 domain binding, the peaks of eight residues (Tyr_60_, Glu_61_, Asp_62_, Trp_65_, Gly_66_, Leu_86_, Gly_87_, and Asp_94_) in the linker region disappeared. These results support wide-scale interactions involving the linker region. In addition, the current study suggested that this interaction should occur through the loop 1 of WW domains, which is not highly conserved in sequence. The difference in peak disappearances in the linker region could be caused by a difference of the loop 1 region between the WW2 and WW3 domains. The tandem WW domains showed higher affinity and sequence specificity for tandem PY motifs compared to the single WW domain [34], which may support the importance of the linker region.

Based on our results, we propose a model of the interaction between human AIP4 and LMP2A (Figure 8). Human AIP4 is a large protein of 862 amino acids and consists of six domains [18]: C2 domain (19–111), WW1 (288–318), WW2 (320–349), WW3 (400–429), WW4 (439–468), and HECT domain (507–860) (Figure 8(B)). Theoretically, two PY motifs of the LMP2A NTD could bind to any of these WW domains. However, there are no or few linking residues between the WW1–WW2 domains and WW3–WW4 domains. This characteristic defines the possible binding of WW domains, as shown in Figure 8(C). Model 1 shows that the two consecutive WW domains (WW1–WW2 or WW3–WW4) interact with LMP2A NTD simultaneously. In this case, two LMP2A molecules could theoretically bind to one hAIP4. In the other model, Model 2, one of the WW1 or WW2 domains binds one PY motif and one of WW3 or WW4 binds the other PY motif of LMP2A. If this is the case, the linking region between WW2 and WW3 should be folded to make the two WW domains spatially adjacent. In both models, the linker region of LMP2A seems to regulate the selectivity of the WW2 domain by interacting with the loop 1 regions of WW domains. Generally, hAIP4 interacts with many proteins containing the PY motif to determine the fate of these proteins in the cell cycle. In any interacting system, the XP grooves of WW domains and PY motifs are almost similar and well conserved. This means that the PY motif itself could not define specificity for WW domains. Thus, hAIP4 needs to specifically regulate binding to a ligand protein, and our model explains this well. In addition, our models have another benefit for additional interactions; the linker region of LMP2A NTD contains the immunoreceptor tyrosine-based activation motif (ITAM, residues 62–88) that binds Syk tyrosine kinase [43]. In our model, ITAM is sufficiently exposed to the water environment, which allows ITAM to be easily accessed by Syk.

Maintaining latency in infected cells and tumorigenesis by LMP2a may be related with the recruiting ability of E3 ligases that provoke the unwanted proteolysis of signal molecules such as protein tyrosine kinases. It is known that Syk is a main molecule in LMP2a signaling [70] and dramatically decreases by LMP2A [71]. As described above, the binding region of Syk is located at the linker region and the mutation of Y74 in the linker region causes loss of functionality of LMP2A in B-cells [72]. Our result showed that Y74 was affected by WW2 binding (Figure 8A), which might support that conformational importance of the linker region for E3 ligase recruiting and substrates binding. In addition, it is noteworthy that the ITAM deletion mutant of LMP2A causes a decrease in cytoplasmic Itch E3 ligase in epithelial cells [23].

## 5. Conclusions

This study provides structural insights into the interaction between two PY motifs of LMP2A NTD and WW domains of the E3 ubiquitin–protein ligase. The XP groove of the WW2 domain may provide a binding pocket for LMP2A NTD PY motifs, and this binding may be optimized by additional interactions between the linker region and WW2 domain. In this process, the hairpin loop region between strands β1 and β2 is likely to act as a sensor for detecting different chemical environments. Further studies of the interactions between PY motifs and WW domains, especially complex structure of two consecutive WW domains and LMP2A NTD, would contribute to understanding the mechanisms and role of the ubiquitination process of LMP2A, which will eventually enable us to elucidate the general mechanisms involving binding between PY motifs and WW domains. 

Recently, proteolysis targeting chimera (PROTAC) techniques have become popular to discover novel drugs, which overcome the traditional small chemical drug. It uses cellular E3 ligase systems to degrade target proteins through physically linking between E3 ligases and target proteins [73]. Our studies on the interaction of AIP4 E3 ligase and its substrate may also be helpful to discover a peptide-mediated PROTAC that is worthy of investigating [74].

## Figures and Tables

**Table 1:** Structural statistics for the ensemble of 20 structures of WW2 domain.

Experimental Constraints	
NOE constraints total	305
Intra-residue (i = j)	114
Sequential (|i-j| = 1)	100
Medium range (1 < |i-j| < 5)	30
Long range (|i-j| ≥ 5)	61
Dihedral constraints	
Φ	15
ψ	16
RMSD from idealized geometry	
Bonds (Å)	0.002 ± 0.00007
Angles (°)	0.3455 ± 0.0062
RMSD to the mean structure (residues 7–16, 20–34)	
#Backbone atoms (N, Cα, CO)	0.41 ± 0.09
#All heavy	0.99 ± 0.11
CNS energy (kcal/mol) ^a^	
E_overall_	55.72 ± 0.97
E_bond_	2.71 ± 0.19
E_angle_	21.44 ± 0.77
E_improper_	2.66 ± 0.31
E_vdw_	20.77 ± 0.96
E_noe_	8.11 ± 0.59
E_cdih_	0.02 ± 0.03
Violations per conformer	
Distance constraints (>0.1 Å)	0
Dihedral angle constraints (>5 Å)	0
van der Waals (<1.6 Å)	0
Ramanchandran plot (%) ^b^	
Most favored region	70.7
Additionally allowed region	20.2
Generously allowed region	8.6
Disallowed region	0.5

^a^ The default parameters and force constants of protein-allhdg.param, and anneal.inp in CNS 1.2 were used for structure calculation. ^b^ PROCHECK-NMR was used for calculation. The ramanchandran analysis was done for the ordered region (residues 7–16 and 20–34) except the unordered loops or tails.

## Data Availability

The AIP4 WW3 domain structure is available in the protein data bank.

## Supplementary Materials

The following are available online at https://www.mdpi.com/2075-1729/11/5/379/s1, Figure S1: Spectral perturbation of the WW2 domain upon the PY peptide binding, Figure S2: Spectral perturbation of the WW2 domain upon the N-PY peptide binding, Figure S3: Spectral perturbation of the WW2 domain upon the C-PY peptide binding.
