# Supplementary material for: Molecular Interactions between Two LMP2A PY Motifs of EBV and WW Domains of E3 Ubiquitin Ligase AIP4
Source: Life (Basel). 2021 Apr 22;11(5):379. doi: 10.3390/life11050379 (PMC8190631; doi:10.3390/life11050379)
Supplement: Supplementary file 1 [file life-11-00379-s001.zip › life-1191704-supplementary.pdf]

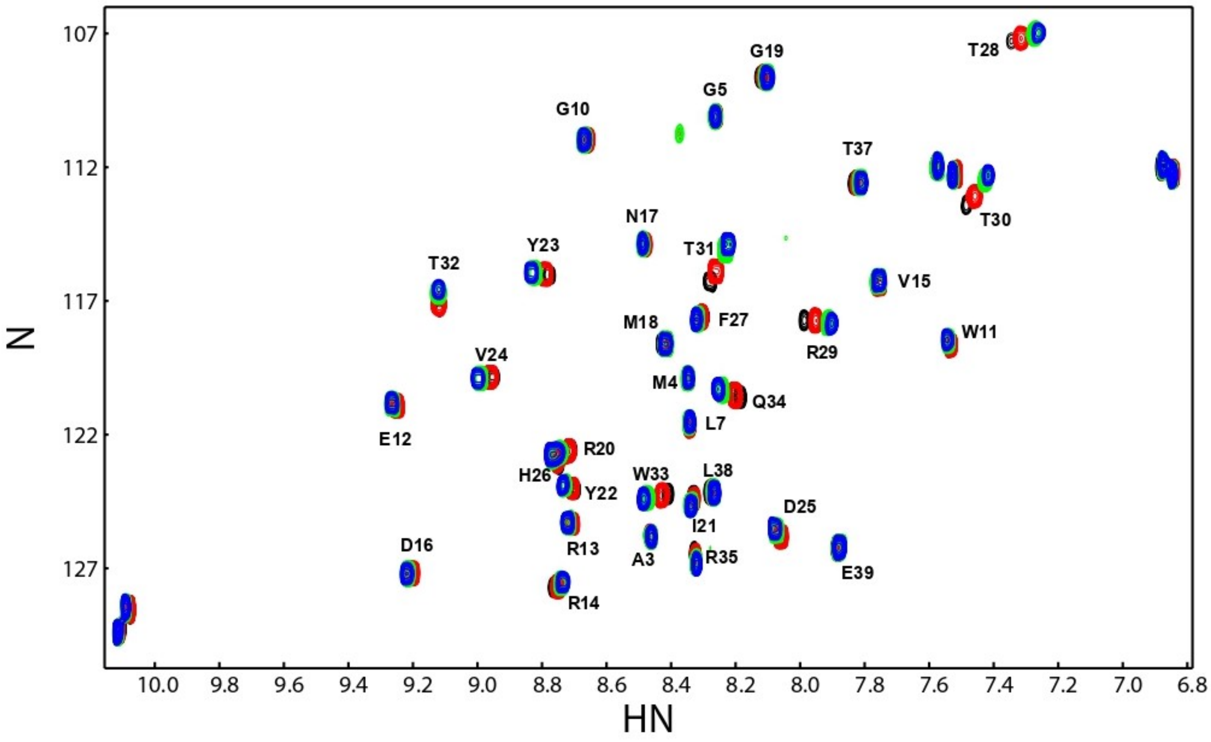

Figure S1. Spectral perturbation of the WW2 domain upon the PY peptide binding. 2 The ^{1}H–^{15}N HSQC spectra of the ^{15}N-labeled WW2 domain upon titration of PY peptide 3 are overlaid. The molar ratios were 1:0 (black), 1:4 (red), 1:16 (green), and 1:32 (blue).

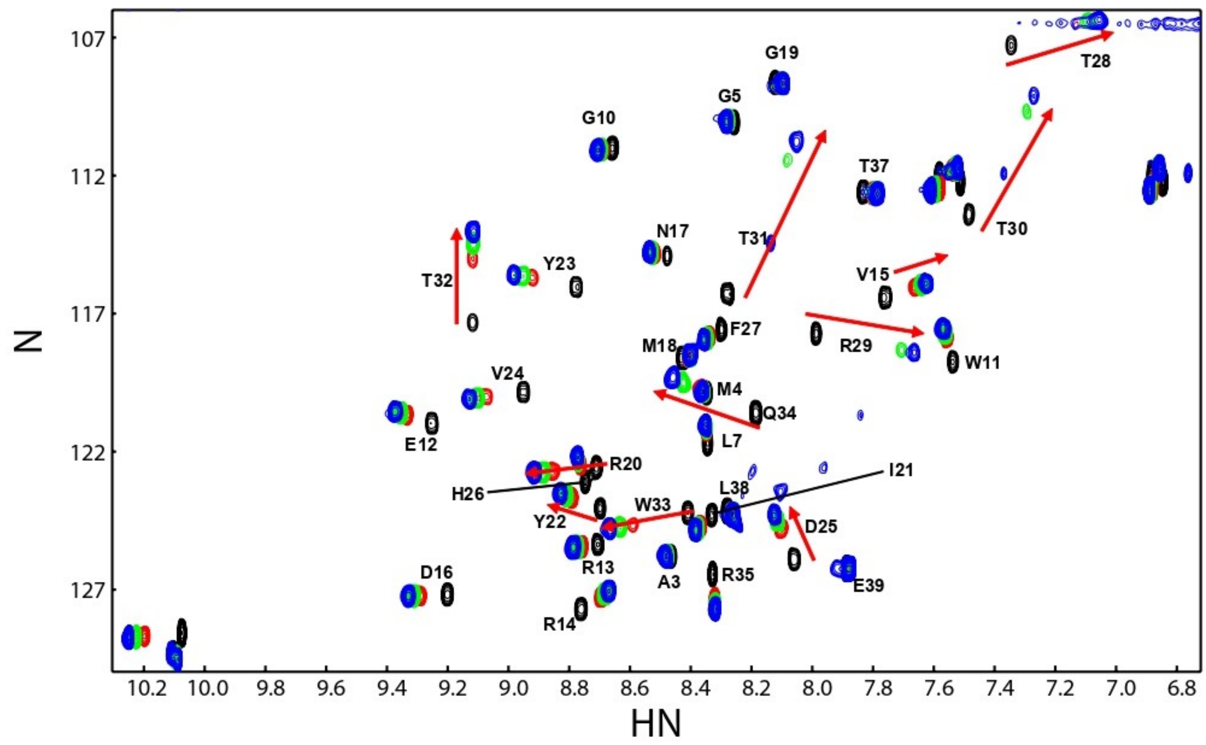

Figure S2. Spectral perturbation of the WW2 domain upon the N-PY peptide 2 binding. The ^{1}H–^{15}N HSQC spectra of the ^{15}N-labeled WW2 domain upon titration of 3 N-PY peptide are overlaid. The molar ratios were 1:0 (black), 1:1 (red), 1:2 (green), and 4 1:4 (blue). The affected residues by the peptide binding were marked with red arrows.

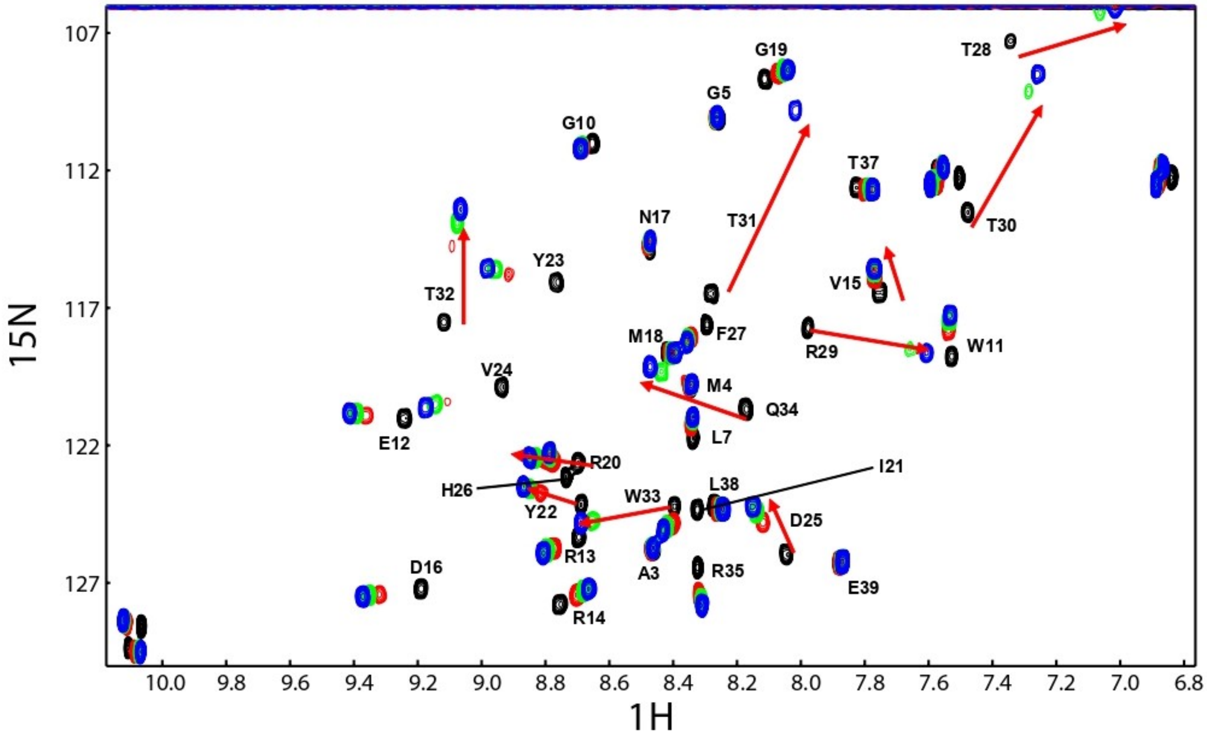

Figure S3. Spectral perturbation of the WW2 domain upon the C-PY peptide 2 binding. The ^{1}H–^{15}N HSQC spectra of the ^{15}N-labeled WW2 domain upon titration of 3 C-PY peptide are overlaid. The molar ratios were 1:0 (black), 1:1 (red), 1:2 (green), and 4 1:4 (blue). The affected residues by the peptide binding were marked with red arrows.
